# Pregnancy and neonatal outcomes after fetal exposure to statins among women with dyslipidemia: a nationwide cohort

**DOI:** 10.1007/s00431-025-06119-3

**Published:** 2025-05-14

**Authors:** Hee Yeon Kay, Ha Young Jang, In-Wha Kim, Jung Mi Oh

**Affiliations:** 1 College of Pharmacy and Research Institute of Pharmaceutical Sciences, Seoul National University, Seoul, Republic of Korea; 2 College of Pharmacy, Gachon University, Incheon, 21936 Republic of Korea; 3 College of Pharmacy, Natural Products Research Institute, Seoul National University, Seoul, 08826 Republic of Korea

**Keywords:** HMG-CoA reductase inhibitors, Drug-induced congenital abnormalities, Pregnancy complications, Retrospective cohort study, Long-term associations

## Abstract

Statin use during pregnancy remains controversial, leading to frequent therapy discontinuation in women with dyslipidemia. This study assessed the association between first-trimester statin exposure and neonatal outcomes, with a focus on long-term developmental effects up to 9 years. Using a nationwide cohort from Korea (2012–2021), we analyzed live births in women with dyslipidemia. Outcomes included congenital malformations and neurodevelopmental disorders. Among 752,351 live births, 218,296 pregnancies met the inclusion criteria after applying exclusions. Of these, 2466 were exposed to statins during the first trimester. Consistent with previous studies, no significant difference in congenital malformations was observed after adjustment (OR 1.00, 95% CI 0.83–1.20). However, subgroup analysis revealed that high-intensity statins were associated with an increased malformation risk (OR 1.47, 95% CI 1.12–1.93). Neurodevelopmental disorders initially appeared more common in the statin-exposed group, but no significant differences were found after adjustment (OR 1.13, 95% CI 0.92–1.41). This finding underscores the long-term associations of statin use during pregnancy in terms of neurodevelopmental outcomes up to 9 years of age. *Conclusion*: This large-scale nationwide cohort study found that statin exposure during early pregnancy did not affect the long-term risk of mental and behavioral disorders in offspring. However, exposure to high-intensity statins was associated with increased risk of congenital malformations. While these findings suggest a potential risk, due to the observational nature of this study, additional studies are required to establish causality.

**Table:** 

**What Is Known:** • Statins, widely used for reducing the risk of atherosclerotic cardiovascular disease, are contraindicated during pregnancy due to concerns about fetal development and potential teratogenic effects. • While early animal studies and some case reports suggested teratogenic risks, recent cohort studies and meta-analyses have not found a significant association between first-trimester statin use and major congenital malformations.	
**What Is New:** • This study, using a large nationwide cohort, found no significant increase in overall congenital malformations after first-trimester statin exposure, but high-intensity statin use was associated with an increased risk. • Long-term neurodevelopmental outcomes up to 9 years showed no significant differences after adjustment, providing novel insights into the safety of statin exposure during pregnancy.

**Supplementary Information:**

The online version contains supplementary material available at 10.1007/s00431-025-06119-3.

## Introduction

Hydroxymethyl glutaryl coenzyme A reductase inhibitors, commonly known as statins, are widely prescribed for their proven efficacy in reducing atherosclerotic cardiovascular disease risks and associated mortality [1, 2]. Statins are among the most commonly used lipid-lowering agents globally, benefiting millions of individuals in both primary and secondary prevention settings. Despite their established safety profile in non-pregnant populations [3], statin use during pregnancy has long been contraindicated due to concerns about potential teratogenic effects. Although this classification has been withdrawn, the U.S. FDA has traditionally used a risk classification system for drug use during pregnancy [4]. Statins were classified as pregnancy category X drugs due to teratogenic effects observed in animal studies, particularly at high doses, including central nervous system and limb anomalies, indicating that their potential risks outweighed any potential benefits [5, 6]. However, findings from human studies have been more heterogeneous, with several recent cohort studies and meta-analyses suggesting that first-trimester exposure may not significantly increase the risk of congenital malformations [7–9].

Pregnancy poses unique physiological challenges, including a natural rise in maternal lipid levels, which supports fetal growth and development [10, 11]. Despite this maternal adaptation, the majority of fetal cholesterol is endogenously synthesized, leading to questions about whether maternal cholesterol modulation via statins exerts a significant impact on fetal outcomes. Moreover, as maternal age and pre-pregnancy comorbidities such as obesity, diabetes, and hypertension increase globally, the prevalence of unplanned pregnancies and incidental statin exposure during early gestation is rising [12, 13]. This growing clinical scenario underscores the importance of understanding the reproductive safety of statins and their implications for fetal development.

While the teratogenicity of statins has been explored to some extent, key gaps remain in the literature [7, 9, 14]. Most studies have focused primarily on congenital malformations, with limited investigation into long-term outcomes. Furthermore, little is known about the role of statin intensity in shaping these outcomes, despite the well-documented dose-dependent effects of statins in animal models. Similarly, sex-specific outcomes, such as the differential susceptibility of male and female fetuses to maternal exposures, remain poorly understood.

A systematic review that included 12 studies suggested that statins might have preventive effects for preeclampsia via endothelial protection and anti-inflammatory effects [15]. Dyslipidemia has been shown to be an independent risk factor for acute myocardial infarction during pregnancy, with an adjusted odds ratio (OR) of 13.11 [16]. Despite the potential beneficial effects of statins during pregnancy, these are not currently recommended for use until more data are collected from human clinical trials.

Therefore, this study aimed to provide a clearer understanding of the risks and benefits associated with statin use during pregnancy. By evaluating both congenital and long-term outcomes, our research offers valuable insights into the safety profile of statins during pregnancy. Additionally, we investigated long-term outcomes, including mental and behavioral disorders, in children exposed to statins in utero.

## Methods

### Data source and study design

The National Health Insurance Service (NHIS)-customized database refers to the health information data which are collected, managed, and maintained by the NHIS to be modified as requested in the purpose of policy and academic research. The NHIS database includes detailed information on demographics, medical history, diagnosis, reimbursements for medical services, medication prescriptions, and treatments. This retrospective cohort study utilized NHIS-customized database to identify all pregnancies in women aged 18 to 44 years with live births between January 1, 2012, and December 31, 2021. The NHIS covers the entire Korean population of approximately 50 million people. The study was approved by the Institutional Review Board of Seoul National University (IRB no. E2212/004–009) and informed consent was waived as all personal information was anonymized.

### Study population

**Fig. 1:**
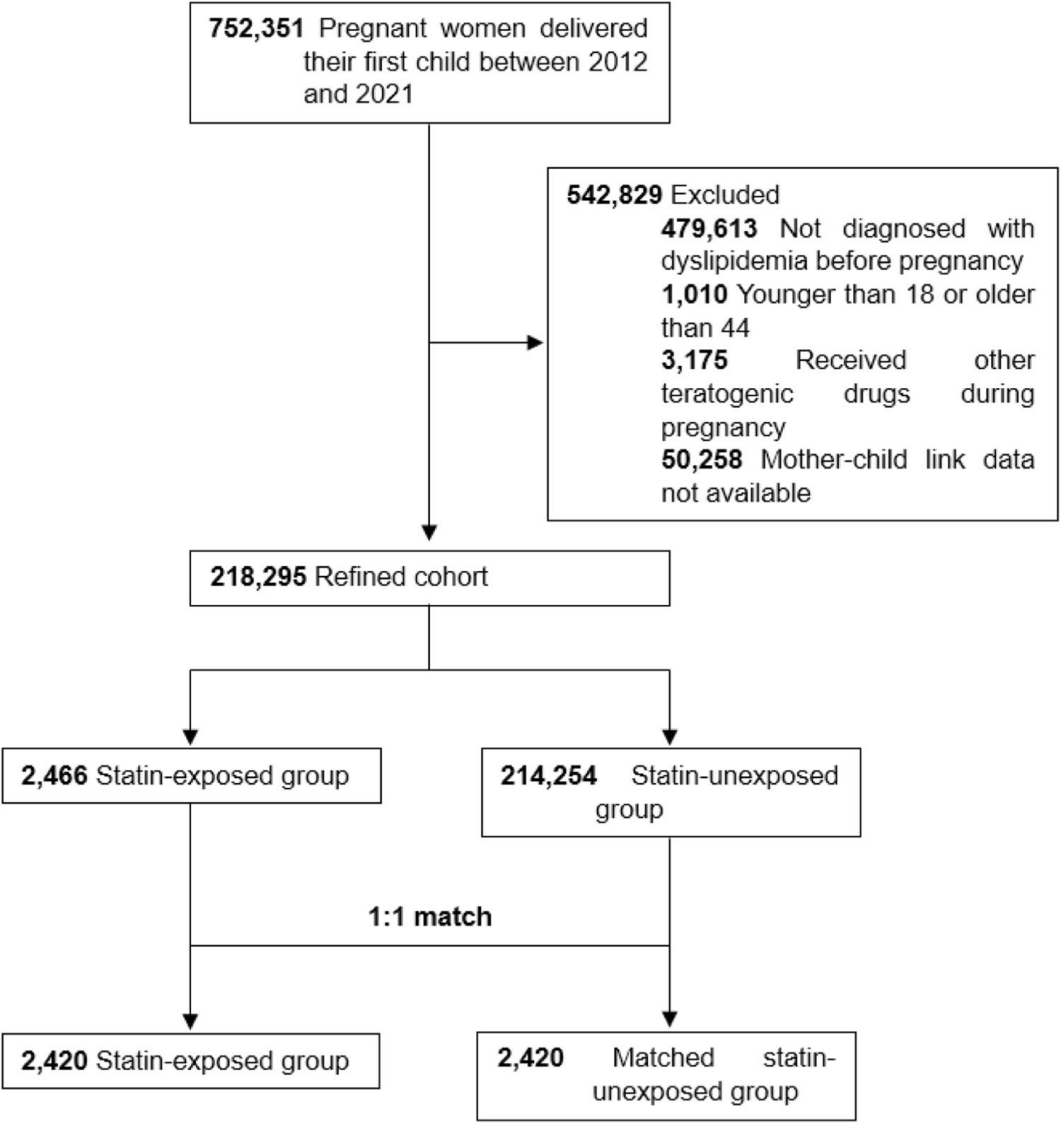
Study flow chart of inclusion and exclusion criteria

Pregnant women with dyslipidemia who gave birth between 2012 and 2021 were identified using International Classification of Diseases, 10 th revision (ICD- 10) codes. Delivery was defined using codes O80.0–O80.4, and dyslipidemia was defined using codes E78.0–E78.9. A total of 752,351 live births were identified (Fig. 1). Out of 752,351 live births, 218,295 pregnancies were included after excluding cases with missing mother–child linkage, exposure to known teratogenic drugs, and multiple births. Details of methods are provided in Supplementary Methods.

### Outcomes

This study evaluated two main outcomes including congenital malformations (ICD- 10 codes Q00–Q99) and long-term neurodevelopmental disorders (ICD- 10 codes F70–F90 [Intellectual disability, Disorders of psychological development, Hyperkinetic disorders]). Long-term neurodevelopmental disorders were identified based on diagnostic codes related to mental and behavioral disorders, with follow-up extending from birth until a maximum of 9 years of age or until the end of the study period in December 2021, whichever came first. As a result, follow-up durations varied depending on the birth year of the child, with children born in earlier years having longer follow-up periods than those born closer to the study endpoint. Given the relatively low event rate and the primary aim of our study to evaluate overall risk rather than incidence over time, standard logistic regression models were used. Details of methods are provided in Supplementary Methods.

### Statistical analyses

Continuous data (age) were presented as mean ± SD, and categorical data (birth year, parity, maternal conditions, concomitant medications, and income level) were presented as numbers and percentages. To minimize confounding, we employed propensity score (PS) matching, a widely used method in observational studies to control for measured confounders and improve comparability between exposure groups. Patients were matched 1:1 using the nearest neighbor approach with a maximum caliper distance of 0.25, according to the recommendations based on previous research [17, 18]. A sub-analysis was conducted based on the intensity of the first statin exposure, duration of exposure, statin administration prior to pregnancy, and the child’s sex. Statin intensity was categorized according to the 2013 the American College of Cardiology and the American Heart Association Guidelines, which classify statins by type and daily dose [19]. Sensitivity analyses were performed to assess the robustness of the primary findings from different perspectives. To address missing data, we followed a complete-case analysis approach. Cases without a mother–child linkage (*n* = 50,258) were excluded to ensure accurate analysis. All statistical analyses were conducted using SAS Enterprise Guide version 7.1 (SAS Institute, Cary, NC, USA). Details of methods are provided in Supplementary Methods.

## Results

### Study population

Table 1 summarizes the demographics of the 216,720 pregnancies; the statin-exposed group had higher rates of medical conditions and concomitant medication use than the unexposed group. Specifically, individuals with diabetes or on diabetes medication were more than six times higher in statin-exposed group. All cohort characteristics were well balanced between the exposed and unexposed groups after PS matching, with SMD < 0.1.

**Table 1:** Baseline characteristics of the cohort

Characteristics	Before PS matching			After PS matching		
	Unexposed (*n* = 214,254)	Statin-exposed (*n* = 2466)	SMD	Unexposed (*n* = 2420)	Statin-exposed (*n* = 2420)	SMD
Mean age (SD), y	32.82 (4.34)	35.28 (4.46)	0.56	35.44 (4.32)	35.27 (4.46)	0.04
Birth year of infants, *n* (%)			0.27			0.09
2012 2013 2014 2015 2016 2017 2018 2019 2020 2021	5385 (2.51) 12,918 (5.96) 18,361 (8.57) 23,423 (10.93) 24,935 (11.64) 25,388 (11.85) 26,104 (12.18) 26,781 (12.50) 25,808 (12.05) 25,151 (11.74)	184 (7.46) 258 (10.46) 242 (9.81) 251 (10.18) 221 (8.96) 246 (9.98) 263 (10.67) 251 (10.18) 275 (11.15) 275 (11.15)		155 (6.4) 271 (11.2) 229 (9.46) 263 (10.87) 197 (8.14) 253 (10.45) 256 (10.58) 250 (10.33) 261 (10.79) 285 (11.78)	180 (7.44) 255 (10.54) 238 (9.83) 246 (10.17) 214 (8.84) 239 (9.88) 260 (10.74) 247 (10.21) 268 (11.07) 273 (11.28)	
Parity, *n* (%)			0.07			0.02
1 2 ≥ 3	119,476 (55.76) 61,379 (28.65) 33,399 (15.59)	1421 (57.62) 641 (25.99) 404 (16.38)		1422 (58.76) 613 (25.33) 385 (15.91)	1398 (57.77) 630 (26.03) 392 (16.20)	
Maternal conditions, *n* (%)						
Diabetes Hypertension	17,297 (8.07) 6569 (3.07)	1287 (52.19) 680 (27.58)	1.10 0.72	1271 (52.52) 654 (27.02)	1266 (52.31) 669 (27.64)	0.00 0.01
Concomitant medications, *n* (%)						
Antidiabetic agents Antihypertensives Antidepressants	3786 (1.77) 2258 (1.05) 2553 (1.19)	1018 (41.28) 533 (21.61) 85 (3.45)	1.10 0.69 0.15	1025 (42.36) 486 (20.08) 66 (2.73)	1003 (41.45) 525 (21.69) 83 (3.43)	0.02 0.04 0.04
Income level, *n* (%)						
First quartile Second quartile Third quartile Fourth quartile	37,534 (18.05) 55,203 (26.54) 72,004 (34.62) 43,240 (20.79)	522 (21.57) 617 (25.50) 833 (34.42) 448 (18.51)	0.10	492 (20.33) 629 (25.99) 858 (35.45) 441 (18.22)	522 (21.57) 617 (25.50) 833 (34.42) 448 (18.51)	0.05

*SD* standard deviation, *SMD* standardized mean differences

### Associations of statins on congenital malformations

The prevalence of overall congenital malformations was 11,717 (5.49%) in the 214,254 pregnancies unexposed to statins, and 252 (10.22%) in the 2466 pregnancies exposed to statins (Table 2). After PS matching to adjust potential confounders, the OR estimates shifted substantially toward a non-significant value (OR 1.00, 95% CI 0.83–1.20). Organ-specific congenital malformations analysis showed similar results.

**Table 2:**
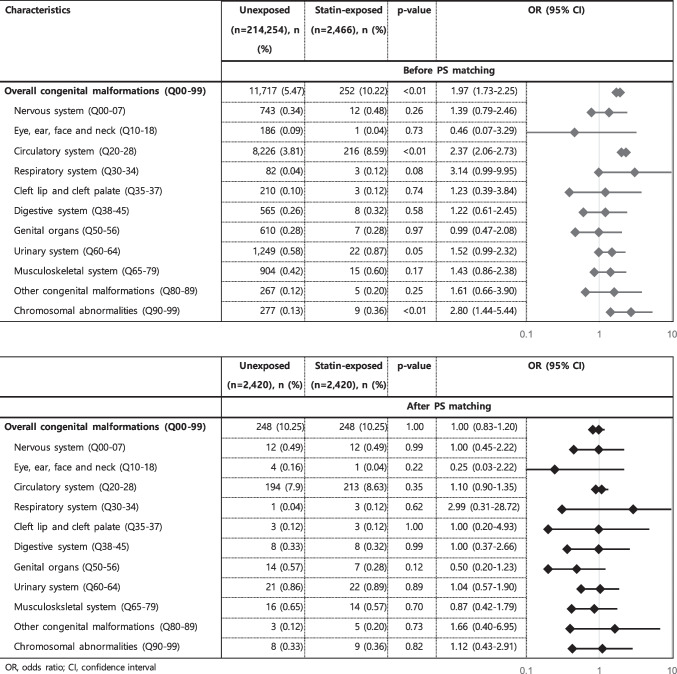
Risks of overall and organ-specific congenital malformations in pregnancies exposed to statins during the first trimester

*OR* odds ratio, *CI* confidence interval

### Long-term associations of statins regarding mental and behavioral disorders

For overall mental and behavioral disorders in children, the OR in the statin-exposure group was significantly higher in the entire cohort. However, after PS matching, the OR was 1.13 (95% CI 0.92–1.41), indicating no significant difference between the two groups (Table 3). Similar results were found for intellectual disability (F70-F79), disorders of psychological development (F80-F89), and behavioral and emotional disorders with onset usually occurring in childhood and adolescence (F90 - 99). The ORs before PS matching were higher in statin-exposure group, but they shifted substantially toward a null value after PS matching. This attenuation suggests that maternal comorbidities, particularly diabetes and hypertension, which were more prevalent in the statin-exposed group, may have contributed to the initial unadjusted associations.

**Table 3:** ORs for mental and behavioral disorders in pregnancies exposed to statins during the first trimester

Variables	Before PS matching				After PS matching			
	Unexposed (*n* = 214,254), *n* (%)	Statin-exposed (*n* = 2466), *n* (%)	*p*-value	OR (95% CI)	Unexposed (*n* = 2420), *n* (%)	Statin-exposed (*n* = 2420), *n* (%)	*p*-value	OR (95% CI)
Mental and behavioral disorders	10,875 (5.08)	196 (7.95)	< 0.01	1.62 (1.39–1.87)	170 (7.02)	191 (7.89)	0.25	1.13 (0.92–1.41)
Intellectual disability (F7)	1171 (0.53)	33 (1.26)	< 0.01	2.41 (1.70–3.41)	30 (1.16)	33 (1.28)	0.70	1.10 (0.67–1.82)
Disorders of psychological development (F8)	8481 (3.82)	157 (5.99)	< 0.01	1.60 (1.36–1.89)	163 (6.32)	154 (5.99)	0.62	0.94 (0.75–1.19)
Behavioral and emotional disorders with onset usually occurring in childhood and adolescence (F9)	9080 (4.09)	162 (6.18)	< 0.01	1.55 (1.32–1.81)	137 (5.31)	157 (6.10)	0.22	1.16 (0.92–1.47)

*OR* odds ratio, *CI* confidence interval

### Impact of statin exposure on low birth weight and preterm birth

The risk of low birth weight in the statin-exposed group remained significantly higher even after PS matching (OR 1.40 [95% CI 1.12–1.74]), with a prevalence of 8.22% compared to 6.03% in the unexposed group (Table 4, Supplementary Table 4). This suggests a persistent association between statin exposure and low birth weight, even after controlling for confounders. In contrast, the initially observed elevated risk of preterm birth in the statin-exposed group (OR 1.44 [95% CI 1.30–1.59] before PS matching) was attenuated after adjustment (OR 1.07 [95% CI 0.93–1.23]). The pre-matching association for preterm birth likely reflects baseline differences between the groups, which were mitigated through PS matching.

**Table 4:** ORs for preterm birth and low birth weight in pregnancies exposed to statins during the first trimester

Variables	Before PS matching				After PS matching			
	Unexposed (*n* = 214,254), *n* (%)	Statin-exposed (*n* = 2466), *n* (%)	*p*-value	OR (95% CI)	Unexposed (*n* = 2420), *n* (%)	Statin-exposed (*n* = 2420), *n* (%)	*p*-value	OR (95% CI)
Preterm birth	32,057 (14.96)	497 (20.15)	< 0.01	1.44 (1.30–1.59)	463 (19.13)	489 (20.21)	0.35	1.07 (0.93–1.23)
Low birth weight	8136 (3.8)	204 (8.27)	< 0.01	2.29 (1.98–2.64)	146 (6.03)	199 (8.22)	< 0.01	1.40 (1.12–1.74)
High birth weight	2607 (1.22)	80 (3.24)	< 0.01	2.72 (2.17–3.41)	76 (3.14)	78 (3.22)	0.87	1.03 (0.75–1.42)

*OR* odds ratio, *CI* confidence interval

### Subgroup analysis

Subgroup analyses were conducted to determine the relationship between the risk of congenital malformations and other outcomes according to the intensity of exposed statins, the duration of statin exposure, whether the statin was administered prior to pregnancy, and the sex of the child. Overall congenital malformations were significantly higher in the high-intensity statin group (OR 1.47 [95% CI 1.12–1.93]) compared to the unexposed group, showing a tendency to increase with statin intensity (Table 5). Low birth weight exhibited a notably higher occurrence solely among boys (OR 1.42 [95% CI 1.04–1.92]). No significant variances were observed in other outcomes based on fetal sex. There were no apparent trends based on statin intensity, exposure duration, or prescriptions before pregnancy.

**Table 5:** Risks of all outcomes in pregnancies exposed to statins during the first trimester: subgroup analysis

Subgroups	Overall congenital malformations, OR (95% CI)	Mental and behavioral disorders, OR (95% CI)	Preterm birth, OR (95% CI)	Low birth weight, OR (95% CI)	High birth weight, OR (95% CI)
Statin intensity					
Unexposed (*n* = 2420) Low-intensity statin (*n* = 82) Moderate-intensity statin (*n* = 2252) High-intensity statin (*n* = 86)	Reference 0.98 (0.89 − 1.08) 1.29 (1.08 − 1.55) 1.47 (1.12 − 1.93)	Reference 1.08 (0.97 − 1.20) 0.94 (0.69 − 1.27) 0.90 (0.57 − 1.43)	Reference 1.04 (0.96 − 1.11) 0.96 (0.80 − 1.16) 0.95 (0.71 − 1.25)	Reference 1.18 (1.05 − 1.32) 1.11 (0.86 − 1.45) 1.18 (0.79 − 1.75)	Reference 1.01 (0.86 − 1.19) 1.15 (0.81 − 1.62) 1.23 (0.73 − 2.05)
Exposure duration					
Unexposed (*n* = 2420) ≤ 30 days (*n* = 730) 30–60 days (*n* = 951) ≥ 60 days (*n* = 739)	Reference 1.05 (0.80 − 1.37) 0.97 (0.85 − 1.10) 1.01 (0.92 − 1.11)	Reference 1.43 (1.07 − 1.91) 1.04 (0.90 − 1.20) 0.97 (0.87 − 1.09)	Reference 1.46 (1.20 − 1.77) 0.96 (0.87 − 1.05) 0.98 (0.91 − 1.05)	Reference 1.55 (1.14 − 2.10) 1.10 (0.94 − 1.27) 1.15 (1.04 − 1.27)	Reference 0.91 (0.56 − 1.49) 1.02 (0.83 − 1.26) 1.04 (0.89 − 1.21)
Prescriptions before pregnancy					
Unexposed (*n* = 2420)	Reference	Reference	Reference	Reference	Reference
Exposed with prescriptions before pregnancy (*n* = 1886)	1.03 (0.85 − 1.26)	1.18 (0.94 − 1.48)	1.15 (0.99 − 1.33)	1.34 (1.06 − 1.69)	0.94 (0.67 − 1.34)
Exposed without prescriptions before pregnancy (*n* = 534)	0.94 (0.80 − 1.11)	0.99 (0.83 − 1.19)	0.90 (0.79 − 1.02)	1.27 (1.07 − 1.50)	1.15 (0.90 − 1.47)
Sex of the child					
Unexposed-male (*n* = 1246)	Reference	Reference	Reference	Reference	Reference
Exposed-male (*n* = 1243)	1.01 (0.79 − 1.30)	1.18 (0.90 − 1.54)	1.13 (0.93 − 1.37)	1.42 (1.04 − 1.92)	1.08 (0.70 − 1.66)
Unexposed-female (*n* = 1161)	Reference	Reference	Reference	Reference	Reference
Exposed-female (*n* = 1166)	0.97 (0.73 − 1.27)	1.07 (0.74 − 1.53)	1.02 (0.83 − 1.26)	1.37 (0.99 − 1.89)	0.97 (0.60 − 1.56)

Although the short-term exposure group (≤ 30 days) showed significantly higher risk of mental and behavioral disorders, preterm birth, and low birth weight, there was no tendency for increased risk of outcomes as the duration of drug exposure increased (Table 5, Supplementary Table 4). The presence or absence of statin prescriptions within 90 days before pregnancy also had no effect. In terms of teratogenicity, mental and behavioral disorders, preterm birth, and high birth weight, the sex of the infant did not demonstrate any notable impact.

### Sensitivity analysis

In sensitivity analyses, the definition of the exposure period was shortened to organogenic period (8 weeks) or the exposure group was restricted to two or more prescriptions. Despite these variations, there was no significant difference in the risk of overall congenital malformations, mental and behavioral disorders, preterm birth, and high birth weight compared to the unexposed group. Only the risk of low birth weight remained significantly higher, consistent with the main analyses (Table 6).

**Table 6:** Fetal risks in pregnancies exposed to statins during the first trimester: sensitivity analysis

Subgroups	Overall congenital malformations, OR (95% CI)	Mental and behavioral disorders, OR (95% CI)	Preterm birth, OR (95% CI)	Low birth weight, OR (95% CI)	High birth weight, OR (95% CI)
Sensitivity analysis: redefined exposure window as up to 8 weeks of pregnancy					
Unexposed (*n* = 2420)	Reference	Reference	Reference	Reference	Reference
Exposed (*n* = 2260)	0.96 (0.79–1.16)	1.17 (0.94–1.46)	1.04 (0.90–1.20)	1.33 (1.06–1.67)	1.06 (0.77–1.46)
Sensitivity analysis: ≥ 2 prescriptions of exposure					
Unexposed (*n* = 2420)	Reference	Reference	Reference	Reference	Reference
Exposed (*n* = 1008)	1.04 (0.93–1.17)	1.14 (1.00–1.30)	1.04 (0.95–1.14)	1.32 (1.16–1.51)	1.17 (0.97–1.42)

## Discussion

This study evaluated maternal statin use during pregnancy and its impact on perinatal outcomes, utilizing a large, nationally representative Korean dataset of over 750,000 pregnancies. Through PS matching and confounder adjustment, we strengthened the reliability of our findings and provided robust risk estimates across different statin intensities and subtypes. While prior studies have generally supported the safety of statins during pregnancy [7, 20, 21], our study contributes additional long-term data on neurodevelopmental outcomes, intensity-dependent risks, and sex-specific effects.

This is the first study to assess the association between maternal statin use and offspring neurodevelopmental disorders. Bateman et al. have reported an initial association between first-trimester statin use and congenital malformations (RR 1.79, 95% CI 1.43–2.23), which became non-significant after confounder adjustment [7]. Our study similarly found that although statin-exposed offspring initially showed higher rates of mental and behavioral disorders, the association disappeared after adjustment (OR 1.13, 95% CI 0.92–1.41). This suggests that the initial association was likely due to maternal comorbidities, particularly diabetes and hypertension, both of which are established risk factors for neurodevelopmental disorders. Diabetes and hypertension may affect placental function, fetal oxygenation, and inflammatory pathways, contributing to altered neurodevelopment. Previous research has linked maternal metabolic disorders to conditions such as autism spectrum disorder, attention-deficit/hyperactivity disorder, and intellectual disability [22, 23]. These findings reinforce the importance of considering maternal health status when evaluating potential risks associated with statin use. Further prospective studies are needed to clarify the mechanisms underlying these associations and determine whether statins themselves play a direct role in neurodevelopmental outcomes.

Consistent with prior studies, our findings show no overall increase in congenital malformations following first-trimester statin exposure after adjustment (OR 1.00, 95% CI 0.83–1.20), suggesting that the initial association was likely driven by maternal comorbidities such as diabetes and hypertension. However, our subgroup analysis revealed that high-intensity statins were associated with an increased risk (OR 1.47, 95% CI 1.12–1.93), suggesting a dose–response effect. This aligns with concerns regarding cholesterol biosynthesis inhibition, which is critical for fetal development. Given that cholesterol’s role in steroid hormone production and placental function, statins may impair placental efficiency, reducing nutrient exchange and fetal growth [24]. Animal studies have demonstrated teratogenic effects of atorvastatin at high doses (10–300 mg/kg), raising concerns about potential developmental toxicity in humans [22]. Given these findings, high-intensity statins should be prescribed with caution, particularly for pregnant individuals with high-risk comorbidities.

Our study classified statins by intensity rather than individual agents. While previous studies suggested lipophilic statins (e.g., atorvastatin, simvastatin) may pose greater teratogenic risks, these associations were no longer significant after PS stratification [7]. Due to the limited number of cases per statin type, detailed subgroup analyses were not feasible. However, given that most statin-exposed pregnancies involved moderate-intensity statins, results for low- and high-intensity groups should be interpreted with caution.

The congenital malformation prevalence in our cohort (5.47%) was higher than European estimates (2.59%) [25] but aligns with increasing trends in Korea (3.44% in 1994 to 5.48% in 2009–2010) [26, 27]. Importantly, our unexposed group had a similar malformation rate before PS matching, suggesting that baseline risk factors (e.g., maternal age, comorbidities) played a significant role. Notably, the statin-exposed group had a higher prevalence (> 10%), supporting concerns about high-intensity statin use. The relatively high prevalence of congenital malformations in this study may be due to the elevated baseline risk in our cohort, which consisted of individuals with dyslipidemia and a higher prevalence of maternal comorbidities such as diabetes and hypertension. Differences in data collection methods, case definitions, and inclusion criteria across studies may also influence reported prevalence rates.

We identified a significant association between statin use and low birth weight, particularly among male infants. This aligns with previous research suggesting sex-specific differential effects of statins in myocardial infarction [28, 29]. Animal studies indicate that male and female placentas respond differently to environmental stressors, with female placentas demonstrating greater adaptability to glucocorticoid exposure, reducing adverse fetal effects [30, 31]. Interestingly, low birth weight risk did not increase with statin intensity, suggesting alternative mechanisms beyond dose-dependent pharmacology. Our subgroup analyses explored the impact of statin intensity, exposure duration, and fetal sex, but these were not pre-specified analyses, potentially leaving room for residual confounding. Future prospective studies are needed to validate these findings.

Unlike some studies reporting higher preterm birth risk among statin-exposed pregnancies [21], our study found no significant association after adjustment. This suggests that statins may not adversely associate with gestational age at delivery. Interestingly, preclinical studies suggest that statins may protect against preterm birth by mitigating pathophysiological mechanisms [32]. Further clinical studies are needed to explore these potential protective effects.

Despite rigorous PS matching, several limitations should be considered. First, exposure misclassification remains a possibility, as prescription records do not confirm actual medication adherence. However, our sensitivity analysis redefined exposure as at least two filled prescriptions, assuming that a refill indicates medication adherence. This approach yielded consistent results, alleviating concerns about misclassification. Second, although maternal comorbidities such as diabetes and hypertension were balanced through PS matching, unmeasured or residual confounding factors such as maternal lifestyle behaviors, disease severity, and medication adherence may have influenced our findings. Third, confounding by indication cannot be ruled out. Women who continued statin use during pregnancy may represent a lower-risk subgroup, while those at higher risk for adverse outcomes may have discontinued statin therapy upon pregnancy confirmation. This selection bias could lead to an underestimation of potential teratogenic risks. Fourth, genetic predisposition was not accounted for in our analysis, which may influence the risk of congenital malformations and neurodevelopmental disorders. Future research integrating genetic data could provide deeper insights into potential hereditary contributions. Fifth, given that the study cohort spans from 2012 to 2021, follow-up durations for offspring varied, leading to potential right-censoring in neurodevelopmental outcome assessments [33]. Children born in earlier years had longer follow-up periods than those born later, which may have influenced the detection of late-onset neurodevelopmental disorders. However, because birth year was included in PS matching, potential bias from differential follow-up duration was minimized. Sixth, selection bias may have occurred if pregnancies were terminated following the detection of severe congenital anomalies, as these cases would not have been captured in our dataset. This may have led to an underestimation of the true incidence of congenital malformations. Future studies incorporating termination records or registry-based data could provide a more comprehensive risk assessment. Seventh, in this study, we excluded patients who had used statins before pregnancy but discontinued them. If a discontinuation comparison group had been included, it could have provided insights into whether residual effects of preconception statin use influence pregnancy outcomes. However, due to dataset limitations, which required estimating the conception date by back-calculating from the delivery date, distinguishing between residual effects and early pregnancy exposure was challenging. Future studies should incorporate a discontinuation group in a prospective design to better address this question. Eighth, detection bias may have influenced neurodevelopmental outcome assessments, as children in the statin-exposed group may have undergone more frequent clinical evaluations due to increased medical surveillance. This heightened attention could have led to higher diagnosis rates of neurodevelopmental disorders, potentially inflating the observed associations. Finally, while diagnostic coding accuracy in administrative databases is generally high, some degree of misclassification or underreporting cannot be excluded. Previous validation studies have demonstrated high reliability for major conditions such as cardiovascular diseases and congenital malformations [34, 35], but potential inaccuracies in less severe or underdiagnosed conditions remain a limitation. Given these constraints, our findings should be interpreted with caution, and further prospective studies with long-term follow-up are warranted to confirm these associations.

In conclusion, this large-scale, nationwide study provides new evidence on the risks associated with maternal statin use, particularly regarding statin intensity, sex-specific outcomes, and long-term neurodevelopmental outcomes. Our findings suggest that first-trimester statin exposure is not associated with increased neurodevelopmental disorder risk, though high-intensity statins may be associated with elevated congenital malformation risk. Given the observational nature of this study, causality cannot be established, and results should be interpreted in the context of potential confounding and selection bias. Further prospective, well-designed studies with long-term follow-up are necessary to provide more definitive clinical guidance on the safety of statins during pregnancy.

## Supplementary Information

Below is the link to the electronic supplementary material.

ESM 1 (DOCX 20.5 KB)

ESM 2 (DOCX 33.8 KB)

## Data Availability

The data that support the findings of this study are available from the database of National Health Insurance Sharing Service (NHISS) (available from https://nhiss.nhis.or.kr/bd/ay/bdaya001iv.do).

## Abbreviations

CVD: Cardiovascular disease
EUROCAT: European Surveillance of Congenital Anomalies
ICD- 10: International Classification of Diseases 10th Revision
NHIS: National Health Insurance Service
OR: Odds ratio
PS: Propensity score
SMD: Standardized mean difference
