# Supplementary material for: Pregnancy and neonatal outcomes after fetal exposure to statins among women with dyslipidemia: a nationwide cohort
Source: Eur J Pediatr. 2025 May 14;184(6):340. doi: 10.1007/s00431-025-06119-3 (PMC12078441; doi:10.1007/s00431-025-06119-3)
Supplement: Supplementary file 1 — (DOCX 20.5 KB) [file 431_2025_6119_MOESM1_ESM.docx]

**Supplementary methods**

**Study Population**

To avoid the potential effect of clustering among siblings, only the firstborn infants of each mother were included. "Firstborn" refers to the first recorded live birth within the study period, including the screening period, from 2011 to 2021. Pregnancies were excluded if they were exposed to other teratogenic drugs (e.g. retinoids, antineoplastic agents, antiepileptics, thalidomide analogues, lithium, misoprostol, mycophenolate, warfarin), as listed in Supplementary Table 1, if mother-child linkage data was unavailable, or if they involved multiple births. The final cohort consisted of 218,295 pregnancies, divided into statin-exposed and unexposed group during the first trimester. Statin-exposed pregnancies were defined as those in which the first statin prescription was filled during the first trimester (weeks 1-12 of gestation). 2,466 pregnancies were exposed to statins during the first trimester. Due to differences in baseline characteristics, a small proportion of statin-exposed pregnancies (n=46) could not be successfully matched and were excluded from the analysis. This group included both cases with a single prescription and those with continued exposure beyond the first trimester. The list of statins included in the analysis is provided in Supplementary Table 2. The last menstrual period was estimated by subtracting 35 weeks (245 days) from the date of birth for preterm deliveries and 39 weeks (273 days) for full-term deliveries, using a previously validated algorithm.[1] The unexposed group consisted of pregnancies with no recorded statin prescriptions at any time, including both the 90 days prior to conception and the entire pregnancy period. In the control group, 1,575 individuals with a history of statin prescriptions within 90 days before pregnancy were excluded. Comorbidities were identified based on diagnostic codes within one year before pregnancy, including diabetes mellitus (E10-14) and hypertension (I10-I13, I15).

**Outcomes**

The presence of congenital malformations was identified through infant records within the first 30 days of life. Major congenital malformations and their subtypes were classified using the European Surveillance of Congenital Anomalies (EUROCAT) classification system, while minor defects were excluded based on the EUROCAT exclusion list.[2] Other outcomes included preterm birth (ICD-10 codes O42 [Premature rupture of membranes before 37 weeks], P07.2 [Extreme immaturity, less than 27 completed weeks], P07.3 [Other preterm infants, 28-36 completed weeks]), low birth weight (ICD-10 codes P07.0 [Extremely low birth weight, less than 999 g], P07.1 [Other low birth weight, 1000-2499 g]), and high birth weight (ICD-10 codes P08.0 [Exceptionally large baby, ≥4500 g], P08.1 [Other heavy for gestational age infants, fetus or infant heavy- or large- for dates regardless of period of gestation, usually implies a birth weight >90^th^ percentile for gestational age or 4000 g or more at term]). Preterm birth was defined as delivery before 37 weeks of gestation and low birth weight was defined as having a birth weight below 2500 g according to the World Health Organization criteria.[3] Congenital malformations were assessed as a binary outcome, defined as the presence or absence of malformations with the first 30 days of life. Since these conditions are typically diagnosed at birth or during the neonatal period, they were analyzed using logistic regression models.

**Statistical analyses**

Continuous variables were compared using Student’s t-test, and categorical variables were compared using the chi-square test.

Covariates included maternal characteristics (age, year of delivery, parity, and socioeconomic status), maternal comorbidities (diabetes mellitus [ICD-10: E10-E14], hypertension [ICD-10: I10-I13, I15]), and concomitant medications (antidiabetic agents, antihypertensives, and antidepressants, as detailed in Supplementary Table 3). Socioeconomic status, a potential confounder in health disparity research, was assessed using the 20-tier insurance premium classification provided by the NHIS. Baseline characteristics, such as maternal age, delivery year, and income level, were collected at the time of delivery, while comorbidities were identified based on diagnostic codes within one year before pregnancy. Concomitant medication use was determined from prescription records within one year before pregnancy.

Standardized mean differences (SMDs) were utilized to compare the baseline characteristics of statin-exposed and statin-unexposed pregnancies. An SMD value of less than 0.1 was considered indicative of balanced characteristics between the two groups. For all outcomes, logistic regression models were applied to estimate the ORs and 95% confidence intervals (CI).

Statin intensity was categorized according to the 2013 the American College of Cardiology and the American Heart Association Guidelines, which classify statins by type and daily dose.[4] High-intensity statins included atorvastatin 40-80 mg and rosuvastatin 20-40 mg. Low-intensity statins included simvastatin 10 mg, pravastatin 10-20 mg, lovastatin 20 mg, fluvastatin 20-40 mg, and pitavastatin 1 mg. The remaining daily doses of statin therapy were classified as moderate-intensity statins.

Sensitivity analyses were performed to assess the robustness of the primary findings from different perspectives. First, considering the first eight weeks of pregnancy as a critical window for organogenesis, we refined the definition of early pregnancy exposure by adjusting the exposure period from 90 days to eight weeks. Second, acknowledging that claims data capture prescriptions rather than actual medication intake, we conducted a sensitivity analysis to evaluate the potential impact of non-adherence. In this analysis, we restricted the statin-exposed group to individuals with at least two recorded prescriptions, under the assumption that repeated dispensing increases the likelihood of actual medication use.

**References**

1. Margulis, A.V., et al., *Algorithms to estimate the beginning of pregnancy in administrative databases.* Pharmacoepidemiol Drug Saf, 2013. **22**(1): p. 16-24.

2. Commission., E. *EUROCAT: European network of population-based registries for the epidemiological surveillance of congenital anomalies*. 18 Jul 2024]; Available from: <https://eu-rd-platform.jrc.ec.europa.eu/eurocat_en>.

3. Organization, W.H., *WHO recommendations for care of the preterm or low birth weight infant*. 2022: World Health Organization.

4. Stone, N.J., et al., *Treatment of blood cholesterol to reduce atherosclerotic cardiovascular disease risk in adults: synopsis of the 2013 American College of Cardiology/American Heart Association cholesterol guideline.* Annals of internal medicine, 2014. **160**(5): p. 339-343.
