# Supplementary material for: Pregnancy and neonatal outcomes after fetal exposure to statins among women with dyslipidemia: a nationwide cohort
Source: Eur J Pediatr. 2025 May 14;184(6):340. doi: 10.1007/s00431-025-06119-3 (PMC12078441; doi:10.1007/s00431-025-06119-3)
Supplement: Supplementary file 2 — (DOCX 33.8 KB) [file 431_2025_6119_MOESM2_ESM.docx]

**Supplementary Table 1. List of known teratogenic drugs**

| **Ingredient code** | **Ingredient name** |
| --- | --- |
| 102301 | acitretin   10mg |
| 118001 | bleomycin hydrochloride   15mg |
| 120630 | busulfan   60mg(6mg/mL) |
| 122701, 122702 | capecitabine   0.15g, 0.5g |
| 123102, 123104, 123130 | carbamazepine   0.2g, 0.3g, 2g(20mg/mL) |
| 123730, 123731, 123732, 123733, 123734, 123735 | carboplatin   50mg(10mg/mL), 0.15g(10mg/mL), 0.45g(10mg/mL), 0.55g(10mg/mL), 0.6g(10mg/mL), 0.65g(10mg/mL) |
| 134530, 134533, 134534 | cisplatin   10mg(0.5mg/mL), 50mg(0.5mg/mL), 50mg(1mg/mL) |
| 134801, 134830 | cladribine   10mg, 10mg(1mg/mL) |
| 139001, 139005 | cyclophosphamide   50mg, 0.5g |
| 139602, 139630, 139631, 139632, 139633, 139634, 139635, 139636, 139637, 139638 | cytarabine   1g, 40mg(20mg/mL), 0.1g(20mg/mL), 0.5g(50mg/mL), 1g(0.1g/mL), 1g(50mg/mL), 2g(50mg/mL), 3g(50mg/mL), 2g(0.1g/mL), 0.1g(50mg/mL) |
| 139901, 139902 | dacarbazine   0.1g, 0.2g |
| 140601 | daunorubicin hydrochloride   20mg |
| 146830 | phenytoin sodium   0.1g(50mg/mL) |
| 148306, 148340, 148341, 148342, 148344, 148346, 148348, 148350, 148351 | docetaxel   0.15g(40mg/mL), 0.12g(20mg/mL), 20mg, 80mg, 20mg(20mg/mL), 20mg(40mg/mL), 80mg(20mg/mL), 80mg(40mg/mL), 0.12g(40mg/mL) |
| 149430 | doxorubicin hydrochloride   10mg(2mg/mL) |
| 149431, 149435 | liposomal doxorubicin hydrochloride (as doxorubicin hydrochloride   20mg(2mg/mL)), 50mg(2mg/mL)) |
| 149432, 149433 | doxorubicin hydrochloride   50mg(2mg/mL), 0.1g(2mg/mL) |
| 152730, 152731 | epirubicin hydrochloride   10mg(2mg/mL), 50mg(2mg/mL) |
| 155101 | estramustine sodium phosphate   0.14g |
| 157102, 157131, 157132 | etoposide   25mg, 0.1g(20mg/mL), 0.15g(20mg/mL) |
| 160101, 160102 | fludarabine phosphate   50mg, 10mg |
| 161430, 161431, 161432 | 5-fluorouracil   0.25g(50mg/mL), 0.5g(50mg/mL), 1g(50mg/mL) |
| 164930, 164931, 164932 | gemcitabine hydrochloride (as gemcitabine   1g(38mg/mL), 0.2g(38mg/mL), 2g(38mg/mL)) |
| 173002 | idarubicin hydrochloride   5mg |
| 173301 | ifosfamide   1g |
| 177430, 177431, 177433, 177435 | irinotecan hydrochloride   40mg(20mg/mL), 0.1g(20mg/mL), 0.2g(20mg/mL), 0.3g(20mg/mL) |
| 178701 | isotretinoin   10mg |
| 181401 | L-asparaginase   10,000Kyowa unit |
| 181403 | L-asparaginase(Erwinia)   10KI.U |
| 184701, 184702 | lithium carbonate   0.3g, 0.15g |
| 189901 | melphalan   2mg |
| 190601 | mercaptopurine hydrate   50mg |
| 192132, 192101, 192134, 192136, 192139, 192141, 192142, 192143, 192144 | methotrexate   2.5mg, 10mg(50mg/mL), 15mg(50mg/mL), 20mg(50mg/mL), 50mg(25mg/mL), 0.5g(25mg/mL), 1g(0.1g/mL), 5g(0.1g/mL), 25mg(50mg/mL) |
| 196301, 196302 | 1% misoprostol powder (as misoprostol   0.1mg, 0.2mg) |
| 196401 | mitomycin C   10mg |
| 196530 | mitoxantrone hydrochloride   23.3mg(2.33mg/mL) |
| 197801, 197802, 197830 | mycophenolate mofetil   0.25g, 0.5g, 34.98g/174.9mL(0.2g/mL) |
| 198001, 198003 | thiotepa   15mg, 0.1g |
| 205803, 205830, 205834 | oxaliplatin   0.15g, 50mg(5mg/mL), 0.1g(5mg/mL) |
| 207830, 207831, 207832, 207833, 207835 | paclitaxel   30mg(6mg/mL), 0.1g(6mg/mL), 0.15g(6mg/mL), 0.2g(6mg/mL), 0.3g(6mg/mL) |
| 211701 | phenobarbital   30mg |
| 211830 | phenobarbital sodium   0.1g(0.1g/mL) |
| 229701, 229703, 229704, 229705, 229706, 229707, 229730, 229731, 229734 | sodium valproate   0.15g, 0.2g, 0.4g, 0.3g, 0.5g, 0.6g, 0.15g(0.1g/mL), 0.3g(0.1g/mL), 9g(60mg/mL) |
| 233401 | sulpiride   0.2g |
| 241901 | topotecan hydrochloride (as topotecan   4mg) |
| 242801, 242802, 242830 | trastuzumab   0.44g, 0.15g, 0.6g(0.12mg/mL) |
| 243001 | tretinoin   10mg |
| 244030 | trifluridine   50mg(10mg/mL) |
| 246901, 246902, 246903 | valproate magnesium   0.5g, 0.2g, 0.3g |
| 247002 | valproic acid   0.5g |
| 247830, 248030, 248031 | vinblastine sulfate   10mg(1mg/mL), 1mg(1mg/mL), 2mg(1mg/mL) |
| 248230, 248231 | vinorelbine tartrate (as vinorelbine   10mg(10mg/mL), 50mg(10mg/mL)) |
| 249103, 249105 | warfarin sodium   2mg, 5mg |
| 309700 | tegafur   0.1g+uracil   0.224g |
| 348101, 348102 | anagrelide hydrochloride (as anagrelide   0.5mg, 1mg) |
| 358202, 358203, 358204 | temozolomide   20mg, 0.1g, 0.25g |
| 412701, 412702, 412703, 412704 | imatinib mesylate (as imatinib   0.1g, 0.2g, 0.4g, 0.3g) |
| 422603, 422630, 422631, 422632 | rituximab   1.4g(0.12g/mL), 0.1g(10mg/mL), 0.5g(10mg/mL), 1.6g(0.12g/mL) |
| 451401, 451402 | mycophenolate sodium (as mycophenolic acid   0.18g, 0.36g) |
| 452400, 452500 | gimeracil   5.8mg+oteracil potassium   19.6mg+tegafur   20mg, gimeracil   7.25mg+oteracil potassium   24.5mg+tegafur   25mg |
| 452801 | belotecan (CKD-602)   2mg |
| 453001 | gefitinib   0.25g |
| 463301, 463302 | bortezomib (PS-341)   3.5mg, 2.5mg |
| 477401, 477402, 477403 | erlotinib hydrochloride (as erlotinib   0.1g, 0.15g, 25mg) |
| 481203, 481230, 481231, 481232, 481234, 481235, 481239 | pemetrexed disodium (as pemetrexed   0.3g, 0.1g (25mg/mL), 0.5g (25mg/mL), 1g (25mg/mL), 0.8g (50mg/mL), 0.8g (25mg/mL), 0.1g (50mg/mL)) |
| 484301, 484302 | azacitidine 0.1g, 0.15g |
| 485501, 485502, 485503 | sirolimus 1mg, 2mg, 0.5mg |
| 485601, 485602, 485603, 485604, 485605, 485606, 485607 | everolimus 0.25mg, 0.5mg, 0.75mg, 1mg, 5mg, 10mg, 2.5mg |
| 485701, 485702 | thalidomide 50mg, 0.1g |
| 487701, 487702, 487703 | sunitinib malate 12.5mg, 25mg, 50mg |
| 488001 | sorafenib tosylate (as sorafenib 0.2g) |
| 495601, 495602 | decitabine 50mg, 40mg |
| 503701 | paclitaxel(albumin-bound) 0.1g |
| 507501 | lapatinib ditosylate (as lapatinib   0.25g) |
| 554330, 554331 | bevacizumab 0.1g (25mg/mL), 0.4g (25mg/mL) |
| 556430 | cetuximab 0.1g(5mg/mL) |
| 568230 | temsirolimus 30mg(25mg/mL) |
| 588201, 588202, 588203, 588204, 588205, 588207 | lenalidomide 10mg, 15mg, 25mg, 5mg, 2.5mg, 20mg |
| 588430 | arsenic trioxide 10mg(1mg/mL) |
| 611801, 611802 | pazopanib hydrochloride (as pazopanib   0.2g, 0.4g) |
| 613901 | cabazitaxel acetone solvate (as cabazitaxel   60mg (40mg/mL)) |
| 614330 | clofarabine 20mg (1mg/mL) |
| 614601, 614602 | bendamustine hydrochloride (as bendamustine   22.7mg, 90.8mg) |
| 617501, 617502 | crizotinib 0.2g, 0.25g |
| 620501 | vemurafenib 0.24g |
| 621001, 621002 | axitinib 1mg, 5mg |
| 621330 | eribulin mesylate (as eribulin   0.88mg (0.44mg/mL)) |
| 623001, 623002, 623003 | ruxolitinib phosphate (as ruxolitinib   5mg, 15mg, 20mg) |
| 623930, 623931 | aflibercept 7.08mg(40mg/mL), 11.12mg (40mg/mL) |
| 624001, 624002 | alitretinoin 10mg, 30mg |
| 624101, 624102 | vandetanib 0.1g, 0.3g |
| 624501 | brentuximab vedotin 50mg |
| 624601 | pertuzumab 0.42g(30mg/mL) |
| 624801 | regorafenib hydrate (as regorafenib   40mg) |
| 626001, 626002 | trastuzumab emtansine (as trastuzumab   0.1g, 0.16g) |
| 626101, 626102, 626103 | afatinib dimaleate (as afatinib   20mg, 30mg, 40mg) |
| 628101 | ibrutinib 140mg |
| 628901 | obinutuzumab 1g(25mg/mL) |
| 633330, 633331 | ipilimumab 50mg(5mg/mL), 0.2g(5mg/mL) |
| 634401 | ceritinib 0.15g |
| 638401, 638402, 638403 | nivolumab 0.1g (10mg/mL), 20mg (10mg/mL), 0.24g (10mg/mL) |
| 639001 | pembrolizumab 0.1g(25mg/mL) |
| 639301, 639302 | ramucirumab 0.1g (10mg/mL), 0.5g (10mg/mL) |
| 643501, 643502, 643503 | olaparib 50mg, 0.15g, 0.1g |
| 645201, 645202 | lenvatinib mesylate (as lenvatinib   4mg, 10mg) |
| 645401, 645402 | trametinib dimethylsulfoxide (as trametinib   2mg, 0.5mg) |
| 647701 | blinatumomab   38.5μg |
| 647801, 647802 | carfilzomib   60mg, 30mg |
| 652501, 652502 | osimertinib mesylate (as osimertinib   40mg, 80mg) |
| 655201, 655202, 655203 | palbociclib   75mg, 0.125g, 0.1g |
| 656201 | alectinib hydrochloride (as alectinib   0.15g) |
| 657701 | atezolizumab   1.2g(60mg/mL) |
| 658801, 658802 | aflibercept 0.1g(25mg/mL), 0.2g(25mg/mL) |
| 663101, 663102 | dabrafenib mesylate (as dabrafenib   50mg, 75mg) |
| 666002 | nanoliposomal irinotecan hydrochloride (as irinotecan 43.3mg (4.33mg/mL)) |
| 666401, 666402, 666403 | cabozantinib (s)-malate (as cabozantinib   60mg, 40mg, 20mg) |
| 667101, 667102 | daratumumab 0.1g (20mg/mL), 0.4g (20mg/mL) |
| 675701, 675702, 675703 | brigatinib 30mg, 90mg, 0.18g |
| 676501 | inotuzumab ozogamicin 1mg |
| 676901, 676902 | durvalumab 0.12g (50mg/mL), 0.5g (50mg/mL) |
| 678801 | avelumab 0.2g(20mg/mL) |
| 678901 | niraparib tosylate (as niraparib 0.1g) |
| 680601, 680602, 680603 | venetoclax 10mg, 50mg, 0.1g |
| 681401, 681402, 681403 | ixazomib citrate (as ixazomib 2.3mg, 3mg, 4mg) |
| 684201 | ribociclib succinate (as ribociclib 0.2g) |
| 686601, 686602, 686603 | abemaciclib 50mg, 0.1g, 0.15g |
| 687801 | gilteritinib fumarate (as gilteritinib 40mg) |
| 688601, 688602 | entrectinib 0.1g, 0.2g |
| 688901, 688902, 688903 | larotrectinib sulfate (as larotrectinib 25mg, 0.1g, 2g (20mg/mL)) |
| 691601, 691602, 691603 | dacomitinib hydrate (as dacomitinib 15mg, 30mg, 45mg) |
| 695101 | lazertinib mesylate (as lazertinib 80mg) |
| 699701, 699702 | lorlatinib 25mg, 0.1g |
| 706201 | tisagenlecleucel 1.2×10^6~6×10^8 |

**Supplementary Table 2. List of statins**

| **Drug name** | **ATC code** | **Drug code** |
| --- | --- | --- |
| simvastatin | C10AA01, C10BA02 | 227801ATB, 227801ATR, 227802ATB, 471100ATB, 507800ATB, 471000ATB |
| lovastatin | C10AA02 | 185801ATB |
| pravastatin | C10AA03, C10BA03 | 216601ATB, 216602ATB, 216603ATB, 216604ATB, 519300ACH |
| fluvastatin | C10AA04 | 162403ATR |
| atorvastatin | C10AA05, C10BX, A10BD, C10BA05, C10BA08 | 111501ATB, 502201ATB, 111502ATB, 502202ATB, 111503ATB, 502203ATB, 111504ATB, 502204ATB, 524000ATB, 524100ATB, 527000ATB, 527100ATB, 688100ATB, 688200ATB, 688300ATB, 688400ATB, 688500ATB, 690400ATB, 690500ATB, 690600ATB, 690700ATB, 472300ATB, 472400ATB, 472500ATB, 518900ATB, 614500ATB, 706300ATB, 706400ATB, 706500ATB, 706600ATB, 706700ATB, 671800ATR, 673800ATR, 671900ATR, 672000ATR, 672100ATR, 633800ATB, 633900ATB, 634600ATB, 634800ATB, 694000ACS, 711200ACS |
| rosuvastatin | C10AA07, C10BX, A10BD, A10BH52, C10BA06, C10BX, C10BA07 | 454001ATB, 454001ATD, 454002ATB, 454002ATD, 454003ATB, 454003ATD, 454005ATB, 526300ATB, 526400ATB, 526500ATB, 526900ATB, 644100ATB, 644200ATB, 653200ATB, 629900ATB, 630000ATB, 630100ATB, 630200ATB, 631600ATB, 631700ATB, 661800ATB, 661900ATB, 662000ATB, 662100ATB, 673700ATB, 663900ATB, 664000ATB, 664100ATB, 664200ATB, 664300ATB, 664400ATB, 671200ATB, 671300ATB, 671400ATB, 671500ATB, 671700ATB, 677000ATB, 677100ATB, 677300ATB, 677400ATB, 677500ATB, 686800ATB, 686900ATB, 679500ATB, 679600ATB, 679700ATB, 680300ATB, 691400ATB, 691500ATB, 683000ATB, 683100ATB, 683200ATB, 691200ATB, 693000ATB, 708900ATB, 684300ATB, 684400ATB, 684500ATB, 684600ATB, 684700ATB, 673900ATB, 674000ATB, 674100ATB, 678600ATB, 525000ATB, 525100ATB, 525200ATB, 525200ATB, 629700ATB, 629800ATB, 654700ATB, 654800ATB, 654900ATB, 655000ATB, 672500ATR, 672600ATR, 672700ATR, 672800ATR, 683300ATR, 683400ATR, 664600ATB, 664700ATB, 664800ATB, 640700ATB, 640800ATB, 640900ATB, 701100ATB, 692000ATB, 692100ATB, 692200ATB, 692300ATB, 692400ATB, 692500ATB, 701900ATB, 702100ATB, 702200ATB, 702000ATB, 709700ATB, 709800ATB, 710000ATB, 710100ATB, 663400ACS |
| pitavastatin | C10AA08, C10BX, C10BA | 470901ATB, 470902ATB, 470903ATB, 634900ATB, 635000ATB, 635100ATB, 635200ATB, 679300ACH, 699400ATB, 699500ATB |

**Supplementary Table 3. List of concomitant medications**

| **Class** | **Drug** |
| --- | --- |
| Antidiabetics | acarbose, glibenclamide, gliclazide, glimepiride, glipizide, gliquidone, tolazamide, chlorpropamide, insulin, metformin, voglibose, rosiglitazone, repaglinide, miglitol, nateglinide, pioglitazone, mitiglinide, vildagliptin, sitagliptin, exenatide, lobeglitazone, dapagliflozin, liraglutide, saxagliptin, linagliptin, gemigliptin, alogliptin, lixisenatide, teneligliptin, empagliflozin, ipragliflozin, anagliptin, dulaglutide, albiglutide, evogliptin, canagliflozin |
| Antihypertensives |  |
| β-blockers | oxoprenolol, pidolol, propranolol, sotalol, nadolol, mepindolol, carteolol, tertatolol, penbutolol, cloranolol, metoprolol, atenolol, acebutolol, betaxolol, bevantolol, bisoprolol, celiprolol, esmolol, nebivolol, labetalol, carvedilol |
| calcium channel blockers | amlodipine, felodipine, isradipine, nicardipine, nifedipine, nimodipine, isoldipine, nitrendipine, lacidipine, nilvadipine, manidipine, barnidipine, lercanidipine, cilnidipine, benidipine, verapamil, gallopamil, diltiazem |
| ACE inhibitors/ARBs | captorpil, enalapril, lisinopril, perindopril, ramipril, quinapril, benazepril, cilazapril, fosinopril, trandolapril, spirapril, delapril, moexipril, temocapril, zofenopril, imidapril, losartan, eprosartan, valsartan, irbesartan, candesartan, telmisartan, olmesartan, fimasartan |
| others | prazosin, indoramin, doxazosin, urapidil, bepridil, methyldopa, hydralazine, clonidine, moxonidine, aliskiren |
| Antidepressants | fluoxetine, paroxetine, escitalopram, sertraline, amitriptyline, nortryptiline, clomipramine, imipramine, venlafaxine, desvenlafaxine, duloxetine, milnacipran |

ACE, angiotensin converting enzyme; ARB, angiotensin receptor blocker

**Supplementary Table 4. Number of outcome events in pregnancies exposed to statins during the first trimester: subgroup analysis and sensitivity analysis**

| **Subgroups** | **Overall congenital malformations, n (%)** | **Mental and behavioural disorders, n (%)** | **Preterm birth, n (%)** | **Low birth weight, n (%)** | **High birth weight, n (%)** |
| --- | --- | --- | --- | --- | --- |
| **Statin intensity**  Unexposed (n=2,420)  Low-intensity statin (n=82)  Moderate-intensity statin (n=2,252)  High-intensity statin (n=86) | 248 (10.25)  7 (8.54)  224 (9.95)  17 (19.77) | 170 (7.02)  5 (6.10)  181 (8.04)  5 (5.81) | 463 (19.13)  18 (21.95)  456 (20.25)  15 (17.44) | 146 (6.03)  8 (9.76)  184 (8.17)  7 (8.14) | 76 (3.14)  2 (2.44)  72 (3.20)  4 (4.65) |
| **Exposure duration**  Unexposed (n=2,420)  ≤30 days (n=730)  30-60 days (n=951)  ≥60 days (n=739) | 248 (10.25)  78 (10.68)  92 (9.67)  78 (10.55) | 170 (7.02)  71 (9.73)  72 (7.57)  48 (6.50) | 463 (19.13)  187 (25.62)  169 (17.77)  133 (18.00) | 146 (6.03)  66 (9.04)  68 (7.15)  65 (8.80) | 76 (3.14)  21 (2.88)  31 (3.26)  26 (3.52) |
| **Prescriptions before pregnancy**  Unexposed (n=2,420)  Exposed with prescriptions before pregnancy (n=1,886)  Exposed without prescriptions before pregnancy (n=534) | 248 (10.25)  199 (10.55)  49 (9.18) | 170 (7.02)  154 (8.17)  37 (6.93) | 463 (19.13)  403 (21.37)  86 (16.10) | 146 (6.03)  149 (7.90)  50 (9.36) | 76 (3.14)  56 (2.97)  22 (4.12) |
| **Sex of the child**  Unexposed-Male (n=1,246)  Exposed-Male (n=1,243)  Unexposed-Female (n=1,161)  Exposed-Female (n=1,166) | 136 (10.91)  137 (11.02)  112 (9.65)  109 (9.35) | 110 (8.83)  127 (10.22)  60 (5.17)  64 (5.49) | 246 (19.74)  270 (21.72)  213 (18.35)  218 (18.70) | 77 (6.18)  106 (8.53)  69 (5.94)  93 (7.98) | 41 (3.29)  44 (3.54)  35 (3.01)  34 (2.92) |
| **Sensitivity analysis: redefined exposure window as up to 8 weeks of pregnancy**  Unexposed (n=2,420)  Exposed (n=2,260) | 248 (10.25)  223 (4.76) | 170 (7.02)  184 (8.14) | 463 (19.13)  446 (19.73) | 146 (6.03)  178 (7.88) | 76 (3.14)  75 (3.32) |
| **Sensitivity analysis: ≥2 prescriptions of exposure**  Unexposed (n=2,420)  Exposed (n=1,008) | 248 (10.25)  111 (11.01) | 170 (7.02)  90 (8.93) | 463 (19.13)  204 (20.24) | 146 (6.03)  102 (10.12) | 76 (3.14)  43 (4.27) |
